# Validation of International Classification of Diseases criteria to identify severe influenza hospitalizations

**DOI:** 10.1111/irv.12931

**Published:** 2022-01-04

**Authors:** Brittney M. Snyder, Megan F. Patterson, Tebeb Gebretsadik, Pingsheng Wu, Tan Ding, Rees L. Lee, Kathryn M. Edwards, Lindsay A. Somerville, Thomas J. Braciale, Justin R. Ortiz, Tina V. Hartert

**Affiliations:** ^1^ Department of Medicine Vanderbilt University Medical Center Nashville Tennessee USA; ^2^ Department of Biostatistics Vanderbilt University Medical Center Nashville Tennessee USA; ^3^ Department of Pediatrics Naval Medical Center Portsmouth Virginia USA; ^4^ Department of Pediatrics Vanderbilt University Medical Center Nashville Tennessee USA; ^5^ Department of Medicine University of Virginia Medical Center Charlottesville Virginia USA; ^6^ Department of Pathology University of Virginia Medical Center Charlottesville Virginia USA; ^7^ Center for Vaccine Development and Global Health University of Maryland School of Medicine Baltimore Maryland USA

**Keywords:** hospitalization, International Classification of Diseases, medical record extraction, severe influenza, validation

## Abstract

In this cohort study of hospitalized patients with linked medical record data, we developed International Classification of Diseases (ICD) criteria that accurately identified laboratory‐confirmed, severe influenza hospitalizations (positive predictive value [PPV] 80%, 95% confidence interval [CI] 71–87%), which we validated through medical record documentation. These criteria identify patients with clinically important influenza illness outcomes to inform evaluation of preventive and therapeutic interventions and public health policy recommendations.

## 1 INTRODUCTION

Influenza control is a global public health priority. However, as of 2014, only 59% of countries had an influenza vaccination policy.^1^ In recommendations for influenza vaccine research and development, the World Health Organization (WHO) stated that “well‐designed studies demonstrating influenza vaccine impact on important public health outcomes,” such as pneumonia and severe illness, “would strengthen the case for their use globally.”^2^ A major limitation to such studies is that the incidence of severe influenza illness is relatively rare, making administrative claims databases across large populations the most efficient data sources for these studies.^3^, ^4^

We aimed to determine the accuracy of International Classification of Diseases (ICD) codes from claims‐based data in identifying severe influenza hospitalizations, which we validated through medical record documentation. Our goal was to address the WHO call for more relevant data on clinically important influenza illness outcomes to inform research, evaluation of preventive and therapeutic interventions, and public health policy recommendations.

## 2 METHODS

We conducted a retrospective cohort study of Tennessee Medicaid (TennCare)^5^ enrollees with severe influenza hospitalizations during the influenza season (defined as October 1 through April 30)^6^ from 1995 through 2017. The study protocol was approved by the Vanderbilt University Medical Center (VUMC) and Tennessee Department of Health Institutional Review Boards. We defined severe influenza hospitalizations as meeting any of following ICD criteria (Table S1):

- Influenza pneumonia: One or more ICD‐9/10 codes for influenza pneumonia
- Influenza with respiratory insufficiency: One or more ICD‐9/10 codes for influenza AND one or more ICD‐9/10 codes for acute respiratory distress/failure, respiratory and circulatory disorders, or continuous mechanical ventilation
- Influenza with other non‐respiratory illness or organ system involvement: One or more ICD‐9/10 codes for influenza AND one or more ICD‐9/10 codes for central nervous system disorders, diseases of the digestive or genitourinary system, shock, sepsis, or in‐hospital death

To access and manually extract medical record information, we restricted our study population to hospital encounters at VUMC. We developed a case report form for medical record extraction with a team of influenza, pulmonary, and critical care experts (Material S1). Two independent physicians extracted medical record information from a random subset of VUMC encounters from unique patients, including laboratory confirmation of influenza virus infection by polymerase chain reaction (PCR), viral culture, and/or rapid antigen test. Pneumonia diagnosis was identified through medical record documentation of pneumonia and/or radiographic findings. Respiratory insufficiency was identified through medical record documentation of apnea, asthma/chronic obstructive pulmonary disease exacerbation, cystic fibrosis exacerbation, mechanical ventilation, oxygen requirement or increased oxygen requirement over baseline, or documented pulmonary function decline. Other non‐respiratory illness or organ system involvement was identified through medical record documentation of acute renal, cardiac, or neurologic deterioration; secondary bacterial infection; sepsis/bacteremia; sickle cell pain crisis; or diabetic ketoacidosis. Hospitalized patients must have had both laboratory confirmation of influenza and medical record documentation of pneumonia, respiratory insufficiency, or other non‐respiratory illness or organ system involvement to be defined as having laboratory‐confirmed, severe influenza based on medical record extraction. We also broadly defined laboratory‐confirmed influenza hospitalizations as encounters with laboratory confirmation of influenza with or without medical record documentation of pneumonia, respiratory insufficiency, or other non‐respiratory illness or organ system involvement based on medical record extraction.

We calculated the positive predictive value (PPV) for (1) laboratory‐confirmed influenza hospitalizations and (2) laboratory‐confirmed, severe influenza hospitalizations by dividing the number of patients with medical record documentation of each of these conditions by the total number of patients identified using our severe influenza hospitalization ICD criteria. We calculated 95% confidence intervals (CIs) for the PPVs using Wilson's formula.^7^ We additionally performed sensitivity analyses to assess the validity of our criteria in identifying severe influenza hospitalizations among children and among individuals with and without underlying respiratory comorbidities. We performed all analyses using R software version 4.0.4 (R foundation for statistical computing, Vienna, Austria). Additional information on methodology can be found in Material S1.

## 3 RESULTS

We identified 25,521 hospitalizations among TennCare enrollees that met the ICD criteria for severe influenza from 1995 through 2017 (Figure S1). Approximately 93% of these hospitalizations occurred during the influenza season. Among these encounters, 1% were hospitalizations at the study hospital. We extracted medical record information from a random subset of 100 hospitalizations from unique patients. More than 50% of these patients were non‐Hispanic, White, female, and non‐smokers (Table S2). The median age at hospitalization was 44 years (interquartile range 20–56 years) (Figure S2), and 45% of patients had at least one respiratory comorbidity.

**FIGURE 1:**
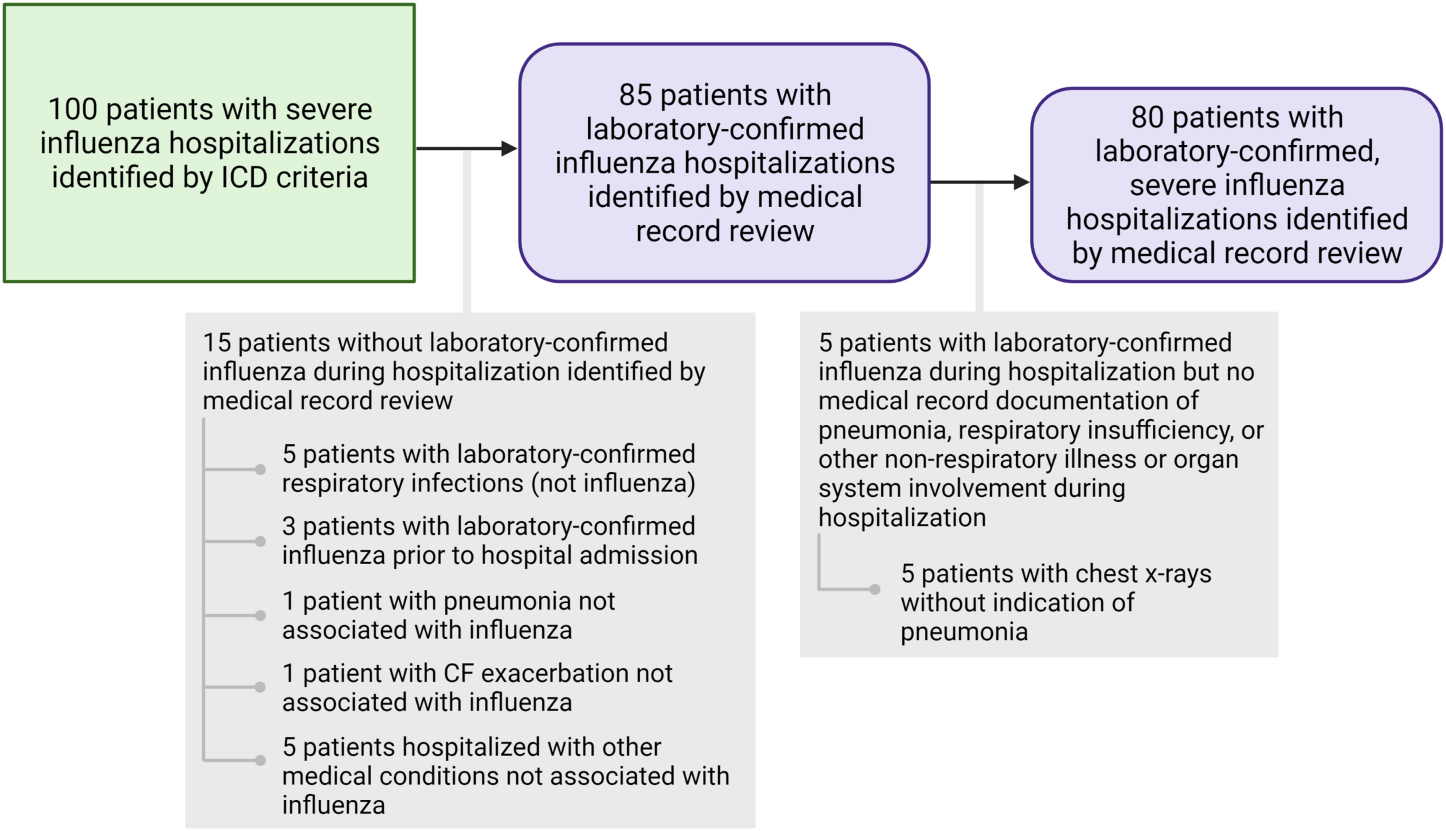
Flow diagram of International Classification of Diseases (ICD) criteria validation with medical record documentation. The green box represents patients identified by our severe influenza hospitalization ICD criteria. The purple boxes represent patients with laboratory‐confirmed influenza and laboratory‐confirmed, severe influenza identified by medical record review. Descriptions of those without laboratory‐confirmed influenza or laboratory‐confirmed, severe influenza identified by medical record review are listed in the grey boxes. (CF, cystic fibrosis). This figure was created with BioRender.com

**FIGURE 2:**
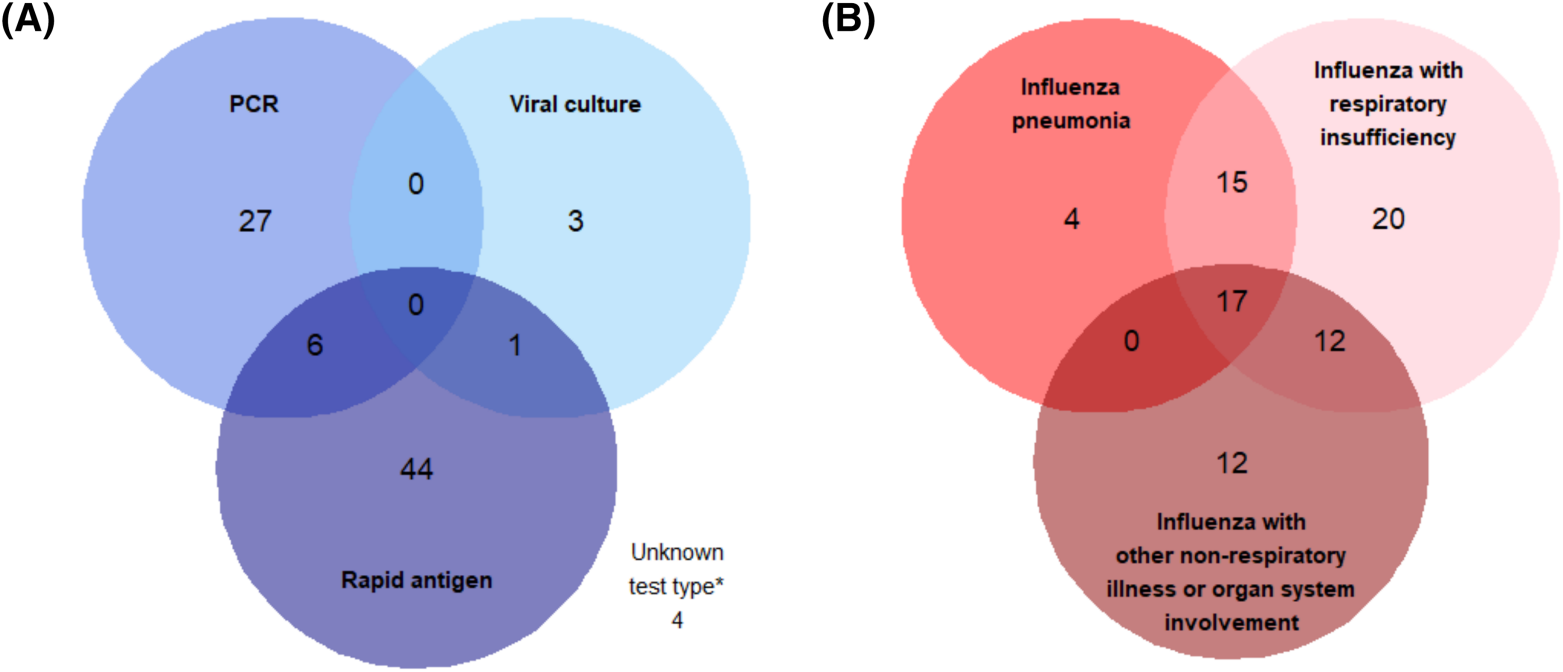
International Classification of Diseases (ICD) criteria accurately identified laboratory‐confirmed, severe influenza hospitalizations at Vanderbilt University Medical Center during influenza seasons, 1995–2017. (A) Positive influenza test type among patients with laboratory‐confirmed influenza identified by medical record review (*n* = 85/100 severe influenza hospitalizations identified by ICD criteria). (B) Type of severe influenza outcomes among patients with laboratory‐confirmed, severe influenza identified by medical record review (*n* = 80/100 severe influenza hospitalizations identified by ICD criteria). (PCR, polymerase chain reaction). *Confirmed influenza cases had documented influenza confirmation only at an outside hospital with an unknown test type

Among the 100 patients with severe influenza hospitalizations identified by our ICD criteria, 85 patients had laboratory‐confirmed influenza identified by medical record review (PPV = 85%, 95% CI 77–91%) (Figure 1). Approximately 32% (*n* = 27) of the patients with laboratory‐confirmed influenza had a positive PCR test, 4% (*n* = 3) had a positive viral culture, 52% (*n* = 44) had a positive rapid antigen test, and 8% (*n* = 7) had a positive result from more than one test type (Figure 2). Approximately 5% (*n* = 4) of these patients had documented influenza confirmation only at an outside hospital with an unknown test type. Of the 15 patients who were identified by our ICD criteria as having severe influenza but did not have laboratory‐confirmed influenza identified by medical record review, five had other laboratory‐confirmed respiratory infections, three had laboratory‐confirmed influenza prior to hospital admission, one had pneumonia not associated with influenza virus infection, one had a cystic fibrosis exacerbation, and five were hospitalized with other medical conditions (Figure 1).

Among the 100 patients with severe influenza identified by our ICD criteria, 80 patients had laboratory‐confirmed, severe influenza identified by medical record review (80% PPV, 95% CI 71–87%) (Figure 1). Among these patients, 5% (*n* = 4) had influenza pneumonia, 25% (*n* = 20) had influenza with respiratory insufficiency, 15% (*n* = 12) had influenza with other non‐respiratory illness or organ system involvement, and 55% (*n* = 44) had more than one of these severe influenza events (Figure 2). All five patients with laboratory‐confirmed influenza during hospitalization but no medical record documentation of pneumonia, respiratory insufficiency, or other non‐respiratory illness or organ system involvement had chest X‐rays without indication of pneumonia (Figure 1).

We performed sensitivity analyses to assess the validity of our criteria in identifying severe influenza hospitalizations among children and individuals with and without underlying respiratory comorbidities. Among the 23 children included in our study population, 19 had laboratory‐confirmed, severe influenza hospitalizations identified by medical record review (83% PPV, 95% CI 63–93%) (Figure S3). Among the 45 patients with underlying respiratory comorbidities and the 55 patients without such conditions, 36 and 44 had laboratory‐confirmed, severe influenza hospitalizations identified by medical record review, respectively (with: 80% PPV, 95% CI 66–89%; without: 80% PPV, 95% CI 68–88%) (Figure S3).

## 4 DISCUSSION

In this cohort study of hospitalized patients with linked medical record data, we developed ICD criteria that accurately identified patients with laboratory‐confirmed, severe influenza hospitalizations, which we validated through medical record documentation. Our criteria also had high PPVs when assessed among children and individuals with and without underlying respiratory comorbidities. Severe influenza is a rare but important outcome, particularly for studies estimating influenza morbidity and vaccine effectiveness.^2^, ^8^, ^9^ Therefore, these criteria could be used to identify patients with clinically important influenza illness outcomes to inform research, evaluation of preventive and therapeutic interventions, and public health policy recommendations.

The accuracy of diagnostic codes in identifying individuals with influenza illness has been previously studied with varying results. In a recent study utilizing a population‐based Canadian cohort, ICD‐10 criteria identified hospitalized patients with laboratory‐confirmed influenza with moderate sensitivity (73%) and high PPV (94%).^10^ Influenza‐specific ICD codes have previously been shown to have sensitivities ranging from 65–86% among children specifically.^3^, ^11^, ^12^ ICD‐9 codes have also been found to accurately estimate the prevalence of influenza pneumonia in hospitalized adults.^13^ To our knowledge, this is the first study to develop criteria to identify severe influenza illness.

Our study was strengthened by our use of a large administrative database to identify severe influenza hospitalizations and linkage with medical records for confirmation. Our detailed case report form was developed and reviewed by a team of experts, and medical record extraction was performed by two independent physicians, which increased the validity of our findings.

We must also recognize some limitations. We restricted our study population to those who had severe influenza during the influenza season, which would have missed cases occurring outside the typical influenza season for the United States. However, as long as the coding pattern is consistent during the influenza season and non‐season, our algorithm should be equally capable of identifying severe influenza events occurring outside the influenza season. As medical record extraction is a tedious endeavor, we were only able to extract information for a subset of patient records, which resulted in small sample sizes for sensitivity analyses and prevented us from having sufficient cases to assess the validity of our ICD criteria among additional subgroups at high risk for influenza hospitalization. Medical record extraction was also only performed at a single academic medical center, which may not reflect documentation and coding at other hospitals.

## 5 CONCLUSIONS

We developed ICD criteria that had high PPV for identifying hospitalized patients with laboratory‐confirmed, severe influenza. As we created these criteria to specifically identify severe influenza‐related hospitalizations for research, they could be applied to identify patients with important influenza public health outcomes, thus addressing the WHO call for more relevant data to inform influenza policy and the impact of preventive and therapeutic interventions.

## CONFLICT OF INTEREST

The authors have no conflicts of interest.

## AUTHOR CONTRIBUTIONS

**Brittney Snyder:** Data curation; formal analysis; investigation; methodology; visualization. **Megan Patterson:** Data curation; formal analysis; investigation; methodology. **Tebeb Gebretsadik:** Data curation; formal analysis; investigation; methodology; visualization. **Pingsheng Wu:** Investigation; methodology. **Tan Ding:** Data curation; investigation; methodology. **Rees Lee:** Investigation; methodology. **Kathryn Edwards:** Investigation; methodology. **Lindsay Somerville:** Investigation; methodology. **Thomas Braciale:** Conceptualization; funding acquisition; investigation; methodology. **Justin Ortiz:** Investigation; methodology; supervision. **Tina Hartert:** Conceptualization; data curation; funding acquisition; investigation; methodology; project administration; resources; supervision; visualization.

### PEER REVIEW

The peer review history for this article is available at https://publons.com/publon/10.1111/irv.12931.

## Supporting information


**Figure S1.** Flow diagram of the study population. This figure was created with BioRender.com.

**Figure S2.** Distribution of age at hospitalization among the study population (n = 100).

**Figure S3.** Type of severe influenza outcomes among A) children with laboratory‐confirmed, severe influenza hospitalizations identified by medical record review (n = 19/23 severe influenza hospitalizations identified by ICD criteria), B) patients with underlying respiratory comorbidities with laboratory‐confirmed, severe influenza hospitalizations identified by medical record review (n = 36/45 severe influenza hospitalizations identified by ICD criteria), and C) patients without underlying respiratory comorbidities with laboratory‐confirmed, severe influenza hospitalizations identified by medical record review (n = 44/55 severe influenza hospitalizations identified by ICD criteria).

**Table S1.** International Classification of Diseases (ICD) codes used to identify severe influenza hospitalizations.
**Table S2.** Clinical and demographic characteristics of the study population (n = 100).

## Data Availability

The data to support the findings of this study were manually extracted from medical records for hospital encounters at Vanderbilt University Medical Center. De‐identified data are available on request from the corresponding author, with Institutional Review Board approval. Data from the Division of TennCare in the Tennessee Department of Finance and Administration were used for case identification. These data are not publicly available due to privacy reasons.
