# Supplementary figures and images for: Validation of International Classification of Diseases criteria to identify severe influenza hospitalizations
Source: Influenza Other Respir Viruses. 2022 Jan 4;16(3):371–5. doi: 10.1111/irv.12931 (PMC8983891; doi:10.1111/irv.12931)

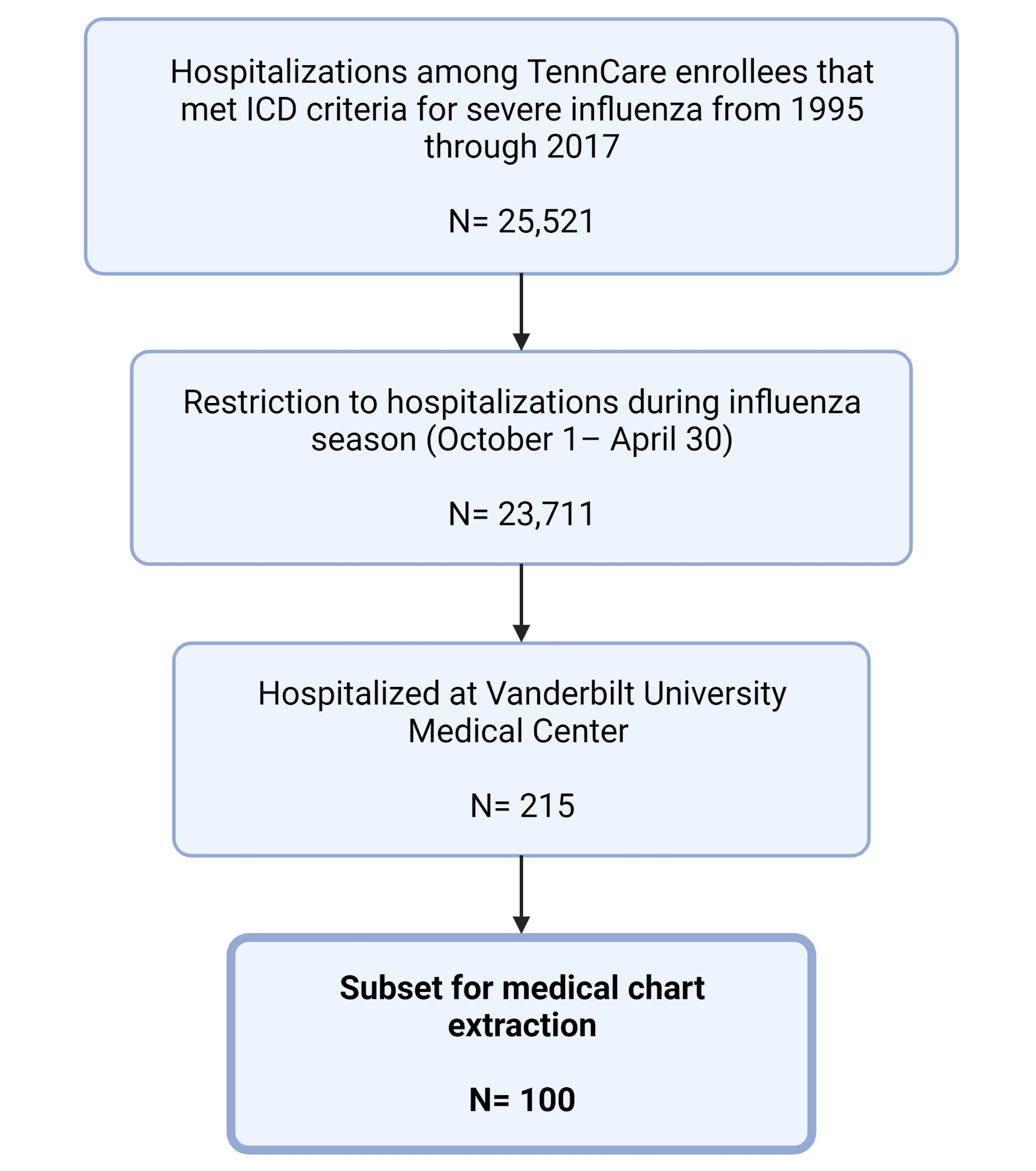

Supplement: Supplementary file 1 — Figure S1. Flow diagram of the study population. This figure was created with BioRender.com. [file IRV-16-371-s004.tiff]

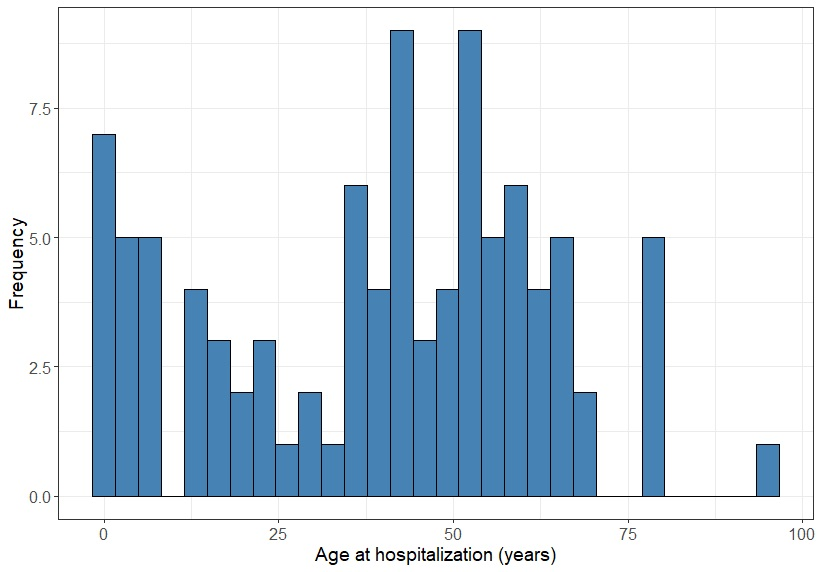

Supplement: Supplementary file 2 — Figure S2. Distribution of age at hospitalization among the study population (n = 100). [file IRV-16-371-s002.tiff]

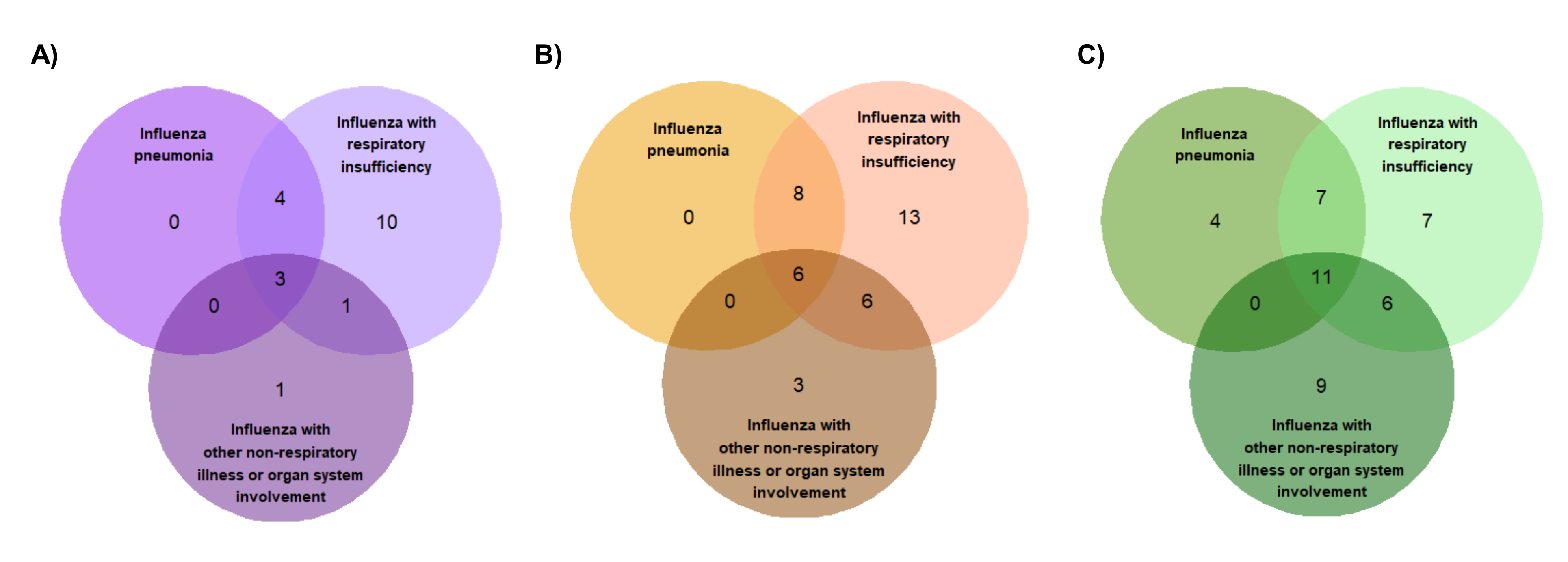

Supplement: Supplementary file 3 — Figure S3. Type of severe influenza outcomes among A) children with laboratory‐confirmed, severe influenza hospitalizations identified by medical record review (n = 19/23 severe influenza hospitalizations identified by ICD criteria), B) patients with underlying respiratory comorbidities with laboratory‐confirmed, severe influenza hospitalizations identified by medical record review (n = 36/45 severe influenza hospitalizations identified by ICD criteria), and C) patients without underlying respiratory comorbidities with laboratory‐confirmed, severe influenza hospitalizations identified by medical record review (n = 44/55 severe influenza hospitalizations identified by ICD criteria). [file IRV-16-371-s001.tiff]
