# Supplementary material for: Validation of International Classification of Diseases criteria to identify severe influenza hospitalizations
Source: Influenza Other Respir Viruses. 2022 Jan 4;16(3):371–5. doi: 10.1111/irv.12931 (PMC8983891; doi:10.1111/irv.12931)
Supplement: Supplementary file 4 — Table S1. International Classification of Diseases (ICD) codes used to identify severe influenza hospitalizations. Table S2. Clinical and demographic characteristics of the study population (n = 100). [file IRV-16-371-s003.docx]

**Validation of International Classification of Diseases criteria to identify severe influenza hospitalizations**

Brittney M. Snyder, Megan F. Patterson, Tebeb Gebretsadik, Pingsheng Wu, Tan Ding,

Rees L. Lee, Kathryn M. Edwards, Lindsay A. Somerville, Thomas J. Braciale, Justin R. Ortiz, Tina V. Hartert

**Supplementary Material**

[Methods 2](#_Toc81395503)

[References 4](#_Toc81395504)

[Supplementary Table 1. 5](#_Toc81395505)

[Supplementary Table 2. 9](#_Toc81395506)

**Severe Influenza Hospitalization Case Report Form.….…..…………………….………………...……...11**

# Methods

We conducted a retrospective cohort study of Tennessee Medicaid (TennCare)^1^ enrollees with severe influenza hospitalizations during the influenza season (defined as October 1 through April 30) from 1995 through 2017. TennCare provides healthcare for approximately 20% of Tennessee’s population; TennCare members are primarily low-income pregnant women and children, individuals with disabilities, and individuals who are elderly.^1^ We restricted our study population to hospitalizations occurring between the months of October-April of each study year to improve specificity by covering time only during influenza season in the United States.^2^

To access and manually extract medical record information, we restricted our study population to hospital encounters at Vanderbilt University Medical Center (VUMC). We developed a case report form for medical record extraction with a team of influenza, pulmonary, and critical care experts (KME, JRO, TVH, and MFP). The case report form included collection of information on patient demographics, clinical data, results of influenza laboratory tests, medical history, diagnostic findings, results of other laboratory tests, treatment, and other risk factors (see **Severe Influenza Hospitalization** **Case Report Form**). Two independent physicians (TVH and MFP) extracted medical record information from a subset of VUMC encounters from 100 unique patients. This subset was chosen randomly and represented multiple study years (**Supplementary Table 2**).

We calculated the positive predictive value (PPV) for 1) laboratory-confirmed influenza hospitalizations and 2) laboratory-confirmed, severe influenza hospitalizations by dividing the number of patients with medical record documentation of each of these conditions by the total number of patients identified using our severe influenza hospitalization ICD criteria. We calculated 95% confidence intervals for the PPVs using Wilson’s formula.^3^ We additionally performed sensitivity analyses to assess the validity of our criteria in identifying severe influenza hospitalizations among 1) children (<18 years at hospitalization), who are at high-risk for influenza hospitalization, 2) individuals with underlying respiratory comorbidities, who are at high-risk for influenza-related morbidity and are a target group for vaccination, and 3) individuals without underlying respiratory comorbidities, among whom severe influenza illness was likely not due to respiratory exacerbation. Respiratory comorbidities were confirmed through medical record review and included asthma, reactive airway disease, chronic obstructive pulmonary disease, asthma-chronic obstructive pulmonary disease overlap syndrome, cystic fibrosis, and other chronic lung disease.

# References

1. Tennessee State Government Division of TennCare. TennCare overview. Available at: <https://www.tn.gov/tenncare/information-statistics/tenncare-overview.html>. Accessed 20 June 2021.
2. Centers for Disease Control and Prevention. Seasonal influenza (flu). Available at: <https://www.cdc.gov/flu/professionals/acip/background-epidemiology.htm>. Accessed 22 June 2021.
3. Brown LD, Cai TT, DasGupta A. Interval Estimation for a Binomial Proportion. *Statistical Science.* 2001;16(2):101-133, 133.

Supplementary Table 1. International Classification of Diseases (ICD) codes used to identify severe influenza hospitalizations.

| **Severe influenza hospitalization event** | **ICD-9 codes** | **ICD-10 codes** |
| --- | --- | --- |
| Influenza pneumonia defined as having one or more ICD-9/10 codes for influenza pneumonia | 487.0 Influenza with pneumonia  488.01 Influenza due to identified avian influenza virus with pneumonia  488.11 Influenza due to identified 2009 H1N1 influenza virus with pneumonia  488.81 Influenza due to identified novel influenza A virus with pneumonia | J09.X1 Influenza due to identified novel influenza A virus with pneumonia  J10.0x Influenza due to other identified influenza virus with pneumonia  J11.0x Influenza due to unidentified influenza virus with pneumonia |
| Influenza with respiratory insufficiency defined as having one or more ICD-9/10 codes for influenza and having one or more ICD-9/10 codes for acute respiratory distress/failure, respiratory and circulatory disorders, or continuous mechanical ventilation | 487.1 Influenza with other respiratory manifestations  487.8 Influenza with other manifestations  488.02 Influenza due to identified avian influenza virus with other respiratory manifestations  488.09 Influenza due to identified avian influenza virus with other manifestations  488.12 Influenza due to identified 2009 H1N1 influenza virus with other respiratory manifestations  488.19 Influenza due to identified 2009 H1N1 influenza virus with other manifestations  488.82 Influenza due to identified novel influenza A virus with other respiratory manifestations  488.89 Influenza due to identified novel influenza A virus with other manifestations  518.5x Pulmonary insufficiency following trauma and surgery  518.81 Acute respiratory failure  518.82 Other pulmonary insufficiency, not elsewhere classified  286.6 Defibrination syndrome  286.9 Other and unspecified coagulation defect  287.4x Secondary thrombocytopenia  287.5 Thrombocytopenia, unspecified  390-459 Diseases of the circulatory system  460-519 (excluding 487.xx and 488.xx) Diseases of the respiratory system  96.7x Other continuous invasive mechanical ventilation | J09.X2 Influenza due to identified novel influenza A virus with other respiratory manifestations  J09.X3 Influenza due to identified novel influenza A virus with gastrointestinal manifestations  J09.X9 Influenza due to identified novel influenza A virus with other manifestations  J10.1 Influenza due to other identified influenza virus with other respiratory manifestations  J10.2 Influenza due to other identified influenza virus with gastrointestinal manifestations  J10.8x Influenza due to other identified influenza virus with other manifestations  J11.1 Influenza due to unidentified influenza virus with other respiratory manifestations  J11.2 Influenza due to unidentified influenza virus with gastrointestinal manifestations  J11.8x Influenza due to unidentified influenza virus with other manifestations  R06.03 Acute respiratory distress  J96.x Respiratory failure, not elsewhere classified  D65 Disseminated intravascular coagulation [defibrination syndrome]  D68.8 Other specified coagulation defects  D68.9 Coagulation defect, unspecified  D69.5x Secondary thrombocytopenia  D69.6 Thrombocytopenia, unspecified  I00-I99 Diseases of the circulatory system  J00-J99 (excluding J09-J11) Diseases of the respiratory system  Z99.1x Dependence on respirator |
| Influenza with other non-respiratory illness or organ system involvement defined as having one or more ICD-9/10 codes for influenza and having one or more ICD-9/10 codes for central nervous system disorders, diseases of the digestive or genitourinary system, shock, sepsis, or in-hospital death | 293.xx Transient mental disorders due to conditions classified elsewhere  348.1 Anoxic brain damage  348.3x Encephalopathy, not elsewhere specified  570 Acute and subacute necrosis of liver  573.4 Hepatic infarction  584.x Acute kidney failure  785.5x Shock without mention of trauma  995.91 Sepsis  995.92 Severe sepsis  798.2 Death occurring in less than 24 hours from onset of symptoms, not otherwise explained  798.9 Unattended death | F09 Unspecified mental disorder due to known physiological condition  G93.1 Anoxic brain damage, not elsewhere classified  G93.4x Other and unspecified encephalopathy  K72.00 Acute and subacute hepatic failure without coma  K76.2 Central hemorrhagic necrosis of liver  K76.3 Infarction of liver  N17.x Acute kidney failure  R57.9 Shock, unspecified  A41.9 Sepsis, unspecified organism  R65.2x Severe sepsis  R99 Ill-defined and unknown cause of mortality |

Supplementary Table 2. Clinical and demographic characteristics of the study population (n=100).

| Characteristic | N or median | % or interquartile range |
| --- | --- | --- |
| Race |  |  |
| White | 61 | 61 |
| Black | 30 | 30 |
| Other | 9 | 9 |
| Ethnicity |  |  |
| Hispanic | 6 | 6 |
| Non-Hispanic | 75 | 75 |
| Unknown | 19 | 19 |
| Sex |  |  |
| Female | 55 | 55 |
| Male | 45 | 45 |
| Smoking status |  |  |
| Current smoker | 22 | 22 |
| Former smoker | 12 | 12 |
| Non-smoker | 60 | 60 |
| Unknown | 6 | 6 |
| Pregnant | 1 | 1 |
| Age at hospitalization | 44 | 20-56 |
| Respiratory comorbidities^†^ |  |  |
| Asthma/reactive airway disease/wheezing | 21 | 21 |
| COPD | 19 | 19 |
| ACOS | 5 | 5 |
| Cystic fibrosis | 3 | 3 |
| Other chronic lung disease | 10 | 10 |
| Year of hospitalization |  |  |
| 1995-1999 | 2 | 2 |
| 2000-2004 | 0 | 0 |
| 2005-2009 | 19 | 19 |
| 2010-2017 | 79 | 79 |

COPD, chronic obstructive pulmonary disease; ACOS, asthma-chronic obstructive pulmonary disease overlap syndrome.

^†^Patients may have had more than one respiratory comorbidity.

| 1 | Chart Review Date | | | | 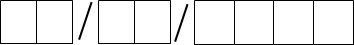 | | | | | | | | | | | | | | | | | | | | |
| --- | --- | --- | --- | --- | --- | --- | --- | --- | --- | --- | --- | --- | --- | --- | --- | --- | --- | --- | --- | --- | --- | --- | --- | --- | --- |
| **SECTION I: PATIENT DEMOGRAPHIC DATA** | | | | | | | | | | | | | | | | | | | | | | | | | |
| 2 | Date of birth: | | | | | | | 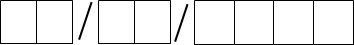  (mm/dd/yy) | | | | | | | | | | | | | | | | | |
| 3 | Race: | | | | | | | American Indian/Alaska Native  Asian  Native Hawaiian/Other Pacific Islander  White | | | | | | | | | | Black  Multiracial  Unknown/ Not Available | | | | | | | |
| 4 | Ethnicity: | | | | | | | Hispanic Non-Hispanic Unknown/Not Available | | | | | | | | | | | | | | | | | |
| 5 | Sex: | | | | | | | Male Female | | | | | | | | | | | | | | | | | |
|  | 5a | Is patient pregnant? | | | | | | Yes | | | | | Weeks pregnant | | | | | | | | No Unknown | | | | |
| 6 | What is patient’s smoking status? | | | | | | | Current Smoker Former Smoker Nonsmoker Unknown/Not Available | | | | | | | | | | | | | | | | | |
| 7 | Is patient exposed to secondhand smoke? | | | | | | | Yes No Unknown | | | | | | | | | | | | | | | | | |
| 8 | Available measurements | | | | | | | Weight Height | | | | | | | | | | | | | | | | | |
|  | 8a | Weight: | | | | | |  English (pounds) **OR** Metric (kilograms)  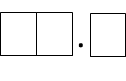  lbs oz kgs | | | | | | | | | | | | | | | | | |
|  | 8b | Height: | | | | | | English (inches) **OR** Metric (cm)  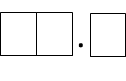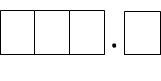  in cm | | | | | | | | | | | | | | | | | |
| 9 | BMI: | | | | | | |  | | | | | | | | | | | | | | | | | |
| 10 | Does this patient have evidence of influenza and/or pneumonia | | | | | | | Yes No END SURVEY | | | | | | | | | | | | | | | | | |
| **SECTION II**: **CLINICAL DATA** | | | | | | | | | | | | | | | | | | | | | | | | | |
| 11 | Number of days of preceding symptoms at presentation: | | | | | | | | | | | | | | | days | | | | | | | | | |
| 12 | Age at onset of illness: | | | | | | | | | | | | | | | years | | | | | | | | | |
|  | 12a | | | If less than one year old, please select age in month: | | | | | <1 mo.  1 mo. | | | 2 mo.  3 mo. | | | | 4 mo.  5 mo. | 6 mo.  7 mo. | | | | | | 8 mo.  9 mo. | | 10 mo.  11 mo. |
| 13 | Signs and symptoms:  (check all that apply) | | | | | Sore throat  Confusion/AMS  Subjective fever  Conjunctivitis | | | Cough  Shortness of breath  Diarrhea  Vomiting | | | | | | | Myalgias  Headache  Seizures  Rhinorrhea  Chills | | | | Malaise, fatigue  Congestion  Other, specify | | | | | |
| 14 | Vitals: | | | | | Fever | | | | | | | | | | Yes No | | | | | | | | | |
|  |  |  |  |  |  | Hypotension (Systolic <90, Diastolic <60) | | | | | | | | | | Yes No | | | | | | | | | |
|  |  |  |  |  |  | Tachycardia (>125 bpm) | | | | | | | | | | Yes No | | | | | | | | | |
|  |  |  |  |  |  | Tachypnea (RR>30) | | | | | | | | | | Yes No | | | | | | | | | |
|  |  |  |  |  |  | Hypothermic | | | | | | | | | | Yes | | | T min | | | | | No | |
| 15 | Was the patient hospitalized? | | | | | | | | | | Yes No Skip to 16 Unknown Skip to 16  No  No | | | | | | | | | | | | | | |
|  | 15a | | Date of admission (mm/dd/yy): | | | | | | | | 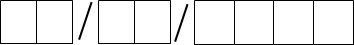  (mm/dd/yy) | | | | | | | | | | | | | | |
|  | 15b | | Date of discharge (mm/dd/yy): | | | | | | | | 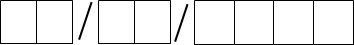  (mm/dd/yy) | | | | | | | | | | | | | | |
|  | 15c | | Was the patient admitted to the intensive care unit? | | | | | | | | | | | Yes No Skip to IH25 Unknown Skip to 16  No | | | | | | | | | | | |
|  |  | | 15c1 | | Date of admission to ICU: | | | | | | | | | 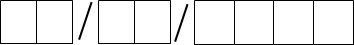  (mm/dd/yy) | | | | | | | | | | | |
|  |  | | 15c2 | | Date of transfer/discharge from ICU: | | | | | | | | | 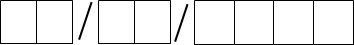  (mm/dd/yy) | | | | | | | | | | | |
|  | 15d | | Did the patient require oxygen supplementation? | | | | | | | | | | | Yes No Skip to 15e Unknown Skip to 15e | | | | | | | | | | | |
|  |  | | 15d1 | | What type(s)? Check all that apply. | | | | | | | | | Nasal cannula/Venturi mask  HFNC  BiPAP  Mechanical ventilation | | | | | | | | Other, specify | | | |
|  |  | | 15d2 | | How long? | | | | | | | | | days | | | | | | | | Unknown | | | |
|  |  | | 15d3 | | On Home O2? | | | | | | | | | Yes No Skip to 15d4 | | | | | | | | | | | |
|  |  | |  | | 15d1a | | Was oxygen requirement while admitted increased from Home baseline? | | | | | | | | | | | | | | | Yes No | | | |
|  |  | | 15d4 | | Maximal oxygen requirement during admission | | | | | | | | | L NC or % FiO_2_  Unknown | | | | | | | | | | | |
|  | 15e | | Did the patient require mechanical ventilation? | | | | | | | | | | | Yes No Skip to 15f Unknown Skip to 15f  No | | | | | | | | | | | |
|  |  | | 15e1 | | How long? | | | | | | | | | days Unknown | | | | | | | | | | | |
|  | 15f | | Did the patient require chest tube? | | | | | | | | | | | Yes No Unknown  No | | | | | | | | | | | |
| 16 | Did the patient die as a result of this illness? | | | | | | | | | | | | | Yes No Unknown  No | | | | | | | | | | | |
| 17 | Did patient have acute comorbid conditions during hospitalization? | | | | | | | | | Yes  No | | | | | Specify | | | | | | | | | | |

| **SECTION III: INFLUENZA TESTING** | | | | | | | | | | | | | | | | | | | | |
| --- | --- | --- | --- | --- | --- | --- | --- | --- | --- | --- | --- | --- | --- | --- | --- | --- | --- | --- | --- | --- |
| 18 | Was influenza testing completed? | | | | | | Yes No Skip to 31 Unknown Skip to 31  No | | | | | | | | | | | | | |
|  | Specimen code and type: | | | | | | | | | | | | | | | | | | | |
|  | 1. Nasopharyngeal swab  2. Nasopharyngeal aspirate  3. Oropharyngeal/throat swab  4. Nasal aspirate/swab  5. Endotracheal aspirate | | | | | 6. Serum  7. Bronchoalveolar lavage specimen (BAL)  8. Sputum  9. Cerebrospinal fluid (CSF) | | | | | | | | 10. Tissue  11. Stool  12. Urine  13. Pleural fluid | | | 14. Peritoneal fluid  24. Pericardial fluid  16. Chest fluid  17. Other | | | |
| **Influenza Test 1** | | | | | | | | | | | | | | | | | | | | |
| 19 | Date collected | | | | | 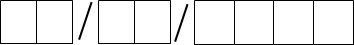 (mm/dd/yyyy) | | | | | | | | | | | | | | |
| 20 | Specimen type code | | | | |  | | | | | | | | | | | | | | |
| 21 | Test type | | | | | RT-PCR/PCR  DFA/IFA | | | Viral culture  HI | | | | Rapid test (antigen)  Immunohistochemistry | | | | | | | Unknown |
| 22 | Results | | | | | Positive Negative Indeterminate | | | | | | | | | | | | | | |
|  | 22a | Influenza Type/Subtype | | | | Flu A  Flu B | | | | Flu A/H1  Flu A/H2B | | | | Flu A unsubtypable  Flu A/H1N1 | | | | Flu A/H3 | | |
| 23 | Source of report | | | | | VUMC testing  No | | | | OSH report/record  No | | | | | | Patient report/record  No | | | | |
| 24 | Was a second influenza test done? | | | | | Yes No Skip to 31  No | | | | | | | | | | | | | | |
| **Influenza Test 2** | | | | | | | | | | | | | | | | | | | | |
| 25 | Date collected | | | | 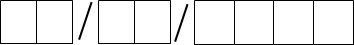 (mm/dd/yyyy)  No | | | | | | | | | | | | | | | |
| 26 | Specimen type code | | | |  | | | | | | | | | | | | | | | |
| 27 | Test type | | | | RT-PCR/PCR  DFA/IFA | | | Viral culture  HI | | | | Rapid test (antigen)  Immunohistochemistry | | | | | | | Unknown | |
| 28 | Results | | | | Positive Negative Indeterminate | | | | | | | | | | | | | | | |
|  | 28a | Influenza Type/Subtype | | | Flu A  Flu B | | | | | | Flu A/H1  Flu A/H2B | | | | | Flu A unsubtypable  Flu A/H1N1 | | | | |
| 29 | Source of report | | | | VUMC testing  No | | | | | | OSH report/record  No | | | | | Patient report/record  No | | | | |
| 30 | Did the patient have other viral testing? | | Yes  No | Was it Positive or Negative Skip to 31 | | | | | | | | | | | | | | | | |
|  |  |  |  | Rhinovirus  No  No  No  No  RSV (A,B)  Adenovirus  No  Coronovirus (C-229E, C- NL63, C-HKU1, C-OC43)  No | | | | | | | | | | | Parainfluenza (1, 2, 3, 4)  No  Mycoplasma Pneumoniae  No  Other, specify  No | | | | | |

| **SECTION IV: MEDICAL HISTORY** | | | | | | | | | | | | | | | | | | | | | | | | | | | | | | | | | | | | | | | | |
| --- | --- | --- | --- | --- | --- | --- | --- | --- | --- | --- | --- | --- | --- | --- | --- | --- | --- | --- | --- | --- | --- | --- | --- | --- | --- | --- | --- | --- | --- | --- | --- | --- | --- | --- | --- | --- | --- | --- | --- | --- |
| 31 | Did the case-patient receive influenza vaccine during season? | | | | | | | | | | | | | | | | | | | | | Yes No Skip to 32 Don’t know Skip to 32  Don;t No | | | | | | | | | | | | | | | | | | |
|  | 31a | | Date if known | | | | | | | | | | | (mm/dd/yy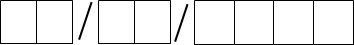) | | | | | | | | | | | | | | | | | | | | | | | | Unknown | | |
| 32 | Does the case-patient have any of the following? | | | | | | | | | | | | | | | | | | | | | | | | | | | | | | | | | | | | | | | |
|  | 32a | | Asthma/Reactive airway disease/wheezing | | | | | | | | | | | | | | | | | | | | | | | | Yes No Unknown  No | | | | | | | | | | | | | |
|  | 32b | | COPD | | | | | | | | | | | | | | | | | | | | | | | | Yes No Unknown  No | | | | | | | | | | | | | |
|  | 32c | | ACOS | | | | | | | | | | | | | | | | | | | | | | | | Yes No Unknown  No | | | | | | | | | | | | | |
|  | 32d | | Cystic fibrosis | | | | | | | | | | | | | | | | | | | | | | | | Yes No Unknown  No | | | | | | | | | | | | | |
|  | 32e | | Other chronic lung disease | | | | | | | | | | | | | | | | | | | | | | | | Yes No Unknown  No | | | | | | | | | | | | | |
|  | 32f | | Chronic heart or circulatory disease | | | | | | | | | | | | | | | | | | | | | | | | Yes No Unknown  No | | | | | | | | | | | | | |
|  | 32g | | Metabolic disease (diabetes mellitus, etc.) | | | | | | | | | | | | | | | | | | | | | | | | Yes No Unknown  No | | | | | | | | | | | | | |
|  | 32h | | Kidney disease | | | | | | | | | | | | | | | | | | | | | | | | Yes No Unknown  No | | | | | | | | | | | | | |
|  | 32i | | Cancer in the last 12 months | | | | | | | | | | | | | | | | | | | | | | | | Yes No Unknown  No | | | | | | | | | | | | | |
|  | 32j | | HIV infection | | | | | | | | | | | | | | | | | | | | | | | | Yes No Unknown  No | | | | | | | | | | | | | |
|  | 32k | | Transplant recipient | | | | | | | | | | | | | | | | | | | | | | | | Yes No Unknown  No | | | | | | | | | | | | | |
|  | 32l | | Chronic corticosteroid therapy | | | | | | | | | | | | | | | | | | | | | | | | Yes No Unknown  No | | | | | | | | | | | | | |
|  | 32m | | Other Immunosuppressive condition | | | | | | | | | | | | | | | | | | | | | | | | Yes No Unknown  No | | | | | | | | | | | | | |
|  | 32n | | Neurological disease | | | | | | | | | | | | | | | | | | | | | | | | Yes No Unknown  No | | | | | | | | | | | | | |
|  | 32o | | Chronic rhinosinusitis (allergic, fungal, etc.) | | | | | | | | | | | | | | | | | | | | | | | | Yes No Unknown  No | | | | | | | | | | | | | |
|  | 32p | | Other chronic diseases | | | | | | | | | | | | | | | | | | | | | | | | Yes No Unknown  No | | | | | | | | | | | | | |
|  | 32q | | History of prematurity | | | | | | | | | | | | | | | | | | | | | | | | Yes No Unknown  No | | | | | | | | | | | | | |
|  | 32r | | Notes | | | | | | |  | | | | | | | | | | | | | | | | | | | | | | | | | | | | | | |
| 33 | Was the patient on chronic medications at the time? | | | | | | | | | | | | | | | | | | | | | | | | | | Yes No Unknown  No | | | | | | | | | | | | | |
|  | 33a | | What medication(s)? Check all that apply. | | | | | | | | | | | | | | | | | | | | | | | | | | | | | | | | | | | | | |
|  |  | | NSAIDs  Tylenol  Montelukast  Antihistamines  Immunosuppressants/Chemotherapy  PPI  Opiates  Systemic corticosteroids  Beta Blockers  ACE inhibitors  ARBs  Ca Channel blocker  Diuretics | | | | | | | | | | | | | | | Statins  Chronic antibiotics  Antiretrovirals/Protease  Inhibitors HIV meds)  HSV antivirals  Metformin  Insulin  Other Diabetes medications  Antidepressants  Anticonvulsants  Antipsychotics  Benzodiazepines  Ambien  Antifungals | | | | | | | | | | | | | | | Antiemetics  Inhaled corticosteroids  Inhaled LABA  Inhaled anticholinergics  Vitamins  BPH meds (alpha-blockers, finasteride)  Laxatives  Biologics  Albuterol, prn  Other, specify  Unknown | | | | | | | |
| 34 | Notes | |  | | | | | | | | | | | | | | | | | | | | | | | | | | | | | | | | | | | | | |
| **SECTION V**: **DIAGNOSTIC FINDINGS** | | | | | | | | | | | | | | | | | | | | | | | | | | | | | | | | | | | | | | | | |
| 35 | Did the patient have any of the following tests? | | | | | | | | | | | | | | | |  | | | | | | | | | | | | | | | | | | | | | | | |
|  | 35a | | Chest X-ray | | | | | | | | | | | | | | Yes  No | | | | | | | | | Normal Abnormal Unknown | | | | | | | | | | | | | | |
|  | 35b | | Chest CT scan | | | | | | | | | | | | | | Yes  No | | | | | | | | | Normal Abnormal Unknown  No | | | | | | | | | | | | | | |
|  | If 35a or 35b = Abnormal 35c. If both are Normal or Unknown Skip to 36 | | | | | | | | | | | | | | | | | | | | | | | | | | | | | | | | | | | | | | | |
|  | 35c | | Was there evidence of pneumonia? | | | | | | | | | | | | | | Yes  No  Unclear  Unknown | | | | | | | | | Check all that apply  No  single lobe multi-lobe unilateral bilateral  No  Explanation if Unclear | | | | | | | | | | | | | | |
|  | 35d | | Did the patient have acute respiratory distress syndrome (ARDS, ARI)? | | | | | | | | | | | | | | | | | | | | | | | | | | | | | | | | Yes No Unknown  No | | | | | |
|  | 35e | | Was there evidence of a pleural effusion? | | | | | | | | | | | | | | | | | | | | | | Yes No Skip to 35f Unknown Skip to 35f | | | | | | | | | | | | | | | |
|  |  | | 35e1 | | | Did they have thoracentesis done? | | | | | | | | | | | | | | | | | | | Yes No Skip to 35f Unknown Skip to 35f | | | | | | | | | | | | | | | |
|  |  | |  | | | 35e1a | | Pleural Fluid Studies: | | | | | | | | | | | | | | | | | Cytology | | | | | | | | | Normal Abnormal | | | | | | |
|  |  |  |  |  |  |  |  |  |  |  |  |  |  |  |  |  |  |  |  |  |  |  |  |  | Culture | | | | | | | | | Normal Abnormal | | | | | | |
|  | 35f | | Was there evidence of progressive infiltrates  (↑size by ≥50% in presence of clinical nonresponse/deterioration)? | | | | | | | | | | | | | | | | | | | | | | | | | | | | | | | Yes No Unknown  No | | | | | | |
|  | 35g | | Was sputum culture done? | | | | | | | | | Yes  No  Unknown | | | | | | | Normal  Abnormal  Unknown | | | | | | | | | Results | | | | | | | | | | | | |
|  | 35h | | Was tracheal/ respiratory culture done? | | | | | | | | | Yes  No  Unknown | | | | | | | Normal  Abnormal  Unknown | | | | | | | | | Results | | | | | | | | | | | | |
|  | 35i | | Other Findings? | | | |  | | | | | | | | | | | | | | | | | | | | | | | | | | | | | | | | | |
| **SECTION VI: GENERAL TESTS** | | | | | | | | | | | | | | | | | | | | | | | | | | | | | | | | | | | | | | | | |
| 36 | | Leukopenia (white blood cell count <5,000 leukocytes/mm3) | | | | | | | | | | | | | | | | | | | | | | | | | | | | | Yes  No  Unknown | | | | | | | | Value | |
| 37 | | Leukocytosis (white blood cell count >10 leukocytes/mm3) | | | | | | | | | | | | | | | | | | | | | | | | | | | | | Yes  No  Unknown | | | | | | | | Value | |
| 38 | | Lymphopenia (total lymphocytes <800/mm3 or lymphocytes <15% of total WBC) | | | | | | | | | | | | | | | | | | | | | | | | | | | | | Yes  No  Unknown | | | | | | | | Value | |
| 39 | | Neutropenia (ANC <1500) | | | | | | | | | | | | | | | | | | | | | | | | | | | | | Yes  No  Unknown | | | | | | | | Value | |
| 40 | | Thrombocytopenia (total platelets <150,000/mm3) | | | | | | | | | | | | | | | | | | | | | | | | | | | | | Yes  No  Unknown | | | | | | | | Value | |
| 41 | | Thrombocytosis (platelets>450,000/mm3) | | | | | | | | | | | | | | | | | | | | | | | | | | | | | Yes  No  Unknown | | | | | | | | Value | |
| 42 | | Elevated BUN (BUN>20) | | | | | | | | | | | | | | | | | | | | | | | | | | | | | Yes  No  Unknown | | | | | | | | Value | |
| 43 | | Elevated Creatinine (≥2 or increase of ≥2 in pt with renal failure) | | | | | | | | | | | | | | | | | | | | | | | | | | | | | Yes  No  Unknown | | | | | | | | Value | |
| 44 | | Lactic acid (Lactate >2.2mmol/L) | | | | | | | | | | | | | | | | | | | | | | | | | | | | | Yes  No  Unknown | | | | | | | | Value | |
| 45 | | Blood culture done? | | | | | | | Yes  No  Unknown | | | | | | Normal  Abnormal  No  Unknown | | | | | | | | | Results | | | | | | | | | | | | | | | | |
| **SECTION VII: TREATMENT** | | | | | | | | | | | | | | | | | | | | | | | | | | | | | | | | | | | | | | | | |
| 46 | | Did the patient receive antiviral medications? | | | | | | | | | | | | | | | | | | | | | | | | | | | | Yes No Unknown  No | | | | | | | | | | |
|  | |  | | Drug | | | | | | | | | | | | | | | | | | | | | | | | | | Date Initiated | | | | | | | | | | |
|  | | 46a | | Oseltamivir(Tamiflu®) | | | | | | | | | | | | | | | | | | | | | | | | | | 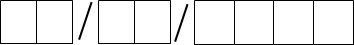 | | | | | | | | | | |
|  | | 46b | | Zanamivir(Relenza®) | | | | | | | | | | | | | | | | | | | | | | | | | | 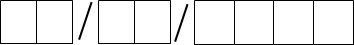 | | | | | | | | | | |
|  | | 46c | | Rimantidine | | | | | | | | | | | | | | | | | | | | | | | | | | 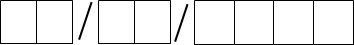 | | | | | | | | | | |
|  | | 46d | | Amantadine | | | | | | | | | | | | | | | | | | | | | | | | | | 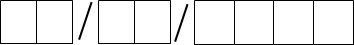 | | | | | | | | | | |
|  | | 46e | | Other (specify) | | | | | | | | | | | | | | | | | | | | | | | | | | 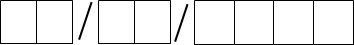 | | | | | | | | | | |
| 47 | | Did the patient receive antibiotic medications? | | | | | | | | | | | | | | | | | | | | | Yes No Skip to 48 Unknown Skip to 48  No | | | | | | | | | | | | | | | | | |
|  | | 47a | | What antibiotic(s)? Check all that apply. | | | | | | | | | | | | | | | | | | | | | | | | | | | | | | | | | | | | |
|  | |  | | Macrolides (azithromycin,  clarithromycin, erythromycin)  Fluoroquinolones (levofloxacin,  moxifloxacin, ciprofloxacin)  Cephalosporins (ceftriaxone,  cefotaxime, cefepime)  Clindamycin | | | | | | | | | | | | Vancomycin  Doxycycline  PCNs (amoxicillin, ampicillin,  piperacillin)  Linezolid  Aztreonam  Carbapenems | | | | | | | | | | | | | | Aminoglycosides (Gentamicin, Amikacin)  Bactrim  Other, specify | | | | | | | | | | |
| 48 | | Did the patient receive antipyretics/anti-inflammatories?  ihtrtatifdone | | | | | | | | | | | | | | | | | | | Yes No Skip to 49 Unknown Skip to 49  No | | | | | | | | | | | | | | | | | | | |
|  | | 48a | | What antipyretics/anti-inflammatories?  Check all that apply | | | | | | | | | | | | | | | | | Aspirin  Other NSAIDs (Ibuprofen,  naproxen, ketorolac, etc.) | | | | | | | | | | | | | | | Acetaminophen  COX-2 selective inhibitors  (Meloxicam, Celecoxib) | | | | |
| 49 | | Did the patient receive vasopressors? | | | | | | | | | | | | | | | | | | | Yes No Skip to 50 Unknown Skip to 50  No | | | | | | | | | | | | | | | | | | | |
|  | | 49a | | What vasopressors?  Check all that apply. | | | | | | | Norepinephrine  Epinephrine  Phenylephrine | | | | | | | | | | Dopamine  Vasopressin  Dobutamine | | | | | | | | | | | Milrinone  Other, specify | | | | | | | | |
| **SECTION VIII: EPIDEMIOLOGIC RISK FACTORS: IF AVAILABLE, ARE ANY OF THE FOLLOWING MENTIONED?** | | | | | | | | | | | | | | | | | | | | | | | | | | | | | | | | | | | | | | | | |
|  | | In the 7 days prior to the illness onset, did the patient… | | | | | | | | | | | | | | | | | | | | | | | | | | | Yes | | | | | | | | No | | | Unknown |
| 50 | | Travel out of the country? | | | | | | | | | | | | | | | | | | | | | | | | | | |  | | | | | | | |  | | |  |
| 51 | | Have close contact with someone with influenza? | | | | | | | | | | | | | | | | | | | | | | | | | | |  | | | | | | | |  | | |  |
| 52 | | Work in a health care facility or setting? | | | | | | | | | | | | | | | | | | | | | | | | | | |  | | | | | | | |  | | |  |
| 53 | | Work in a child care facility or setting? | | | | | | | | | | | | | | | | | | | | | | | | | | |  | | | | | | | |  | | |  |
| 54 | | Have contact with someone with pneumonia or flu like illness? | | | | | | | | | | | | | | | | | | | | | | | | | | |  | | | | | | | |  | | |  |
|  | | Does this patient have: | | | | | | | | | | | | | | | | | | | | | | | | | | | | | | | | | | | | | | |
|  | | 54a | | Definite Influenza | | | | | | | | | Yes  Check all that apply  Then Skip to 54b | | | | | | | PCR  Culture  Rapid antigen | | | | | | | | | | | | | | | | | | | | No  Unknown |
|  | |  | | 54a1 | | Probable Influenza | | | | | | | | | | | | | | Yes No Unknown | | | | | | | | | | | | | | | | | | | | |
|  | | 54b | | Pneumonia | | | | | | | | | Yes  No  Unclear  Unknown | | | | | | | Check all that apply  Clinically diagnosed pneumonia  CXR findings c/w pneumonia  CT findings c/w pneumonia  Initially treated for PNA then discontinued  Other | | | | | | | | | | | | | | | | | | | | |
|  | | 54c | | Respiratory insufficiency | | | | | | | | | Yes  Check all that apply | | | | | | | Apnea  Asthma/COPD exacerbation  CF exacerbation  Mechanical ventilation  Oxygen requirement or increased oxygen  requirement over baseline  FEV1 decline  Other | | | | | | | | | | | | | | | | | | | | No  Unknown |
|  | | 54d | | Other non-respiratory organ system failure | | | | | | | | | Yes  Check all that apply | | | | | | | Renal involvement  Cardiac involvement  Neurologic involvement (seizures, CVAs, etc.)  Sinusitis  Colitis  Sepsis/Bacteremia  Sickle cell pain crisis  Diabetic ketoacidosis  Other | | | | | | | | | | | | | | | | | | | | No  Unknown |
|  | | 54e | | If patient has neither definite nor probable influenza, list the diagnosis | | | | | | | | | | | | |  | | | | | | | | | | | | | | | | | | | | | | | |
| 55 | | Notes | | |  | | | | | | | | | | | | | | | | | | | | | | | | | | | | | | | | | | | |
